# Left Ventricular Flow Distribution as a Novel Flow Biomarker in Atrial Fibrillation

**DOI:** 10.3389/fbioe.2021.725121

**Published:** 2021-11-25

**Authors:** Hansuk Kim, Hana Sheitt, Stephen B. Wilton, James A. White, Julio Garcia

**Affiliations:** ^1^ Biomedical Engineering Graduate Program, University of Calgary, Calgary, AB, Canada; ^2^ Stephenson Cardiac Imaging Centre, University of Calgary, Calgary, AB, Canada; ^3^ Libin Cardiovascular Institute, University of Calgary, AB, Calgary, Canada; ^4^ Department of Cardiac Sciences, University of Calgary, Calgary, AB, Canada; ^5^ Department of Radiology, University of Calgary, Calgary, AB, Canada; ^6^ Alberta Children’s Hospital Research Institute, University of Calgary, Calgary, AB, Canada

**Keywords:** 4D-flow MRI, flow distribution, magnetic resoance imaging, direct flow, arial fibrillation

## Abstract

**Introduction:** Four-dimensional (4-D) flow cardiac magnetic resonance imaging can be used to elegantly describe the hemodynamic efficiency of left ventricular (LV) flow throughout the cardiac cycle. Patients with nonvalvular paroxysmal atrial fibrillation (PAF) may have occult LV disease. Flow distribution analysis, based on 4-D flow, may unmask the presence of LV disease by assessing flow components: direct flow, retained flow, delayed ejection, and residual volume. This study aimed to identify LV hemodynamic inefficiencies in patients with PAF and normal systolic function. We hypothesized that the fraction of direct flow to the total end-diastolic volume would be reduced in patients with PAF compared with controls.

**Methods:** We used 4-D LV flow component analysis to compare hemodynamics in 30 healthy controls and 50 PAF patients in sinus rhythm.

**Results:** PAF subjects and healthy controls had similar LV mass, volume, and ejection fraction. Direct flow was lower in the PAF group than in the controls (44.5 ± 11.2% vs. 50.0 ± 12.2%, *p* = 0.042) while delayed ejection was higher in the PAF group (21.6 ± 5.6% vs. 18.6 ± 5.7%, *p* = 0.022).

**Conclusion:** PAF patients demonstrated a relative reduction in direct flow and elevation in delayed ejection.

## Introduction

Atrial fibrillation (AF) is a growing epidemic affecting approximately 37.6 million individuals worldwide, and this has increased 33% during the last 20 years (Lippi et al., 2021) and is a significant contributor to disability-adjusted life-years in the elderly (Abbafati et al., 2020). Paroxysmal AF (PAF) is typically initiated by premature atrial ectopic beats originating from the pulmonary veins but is maintained by altered atrial refractoriness related to tissue remodeling (Nattel et al., 2014). In the absence of valvular heart disease, the latter is commonly suspected to be related to abnormal loading of the left atrium (LA) during diastole from apparent or occult left ventricular (LV) dysfunction.

Cardiac magnetic resonance (CMR) is the gold standard for the volumetric evaluation of chamber structure and function (Kawel-Boehm et al., 2015). The more recent availability of four-dimensional (4-D) flow magnetic resonance imaging (MRI) provides a unique ability to assess complex 3-D blood flow patterns *in vivo* and may facilitate gaining insights into complex normal and altered intra-cardiac hemodynamics otherwise overlooked by standard 2-D imaging techniques (Markl et al., 2016; Garcia et al., 2020).

**FIGURE 1:**
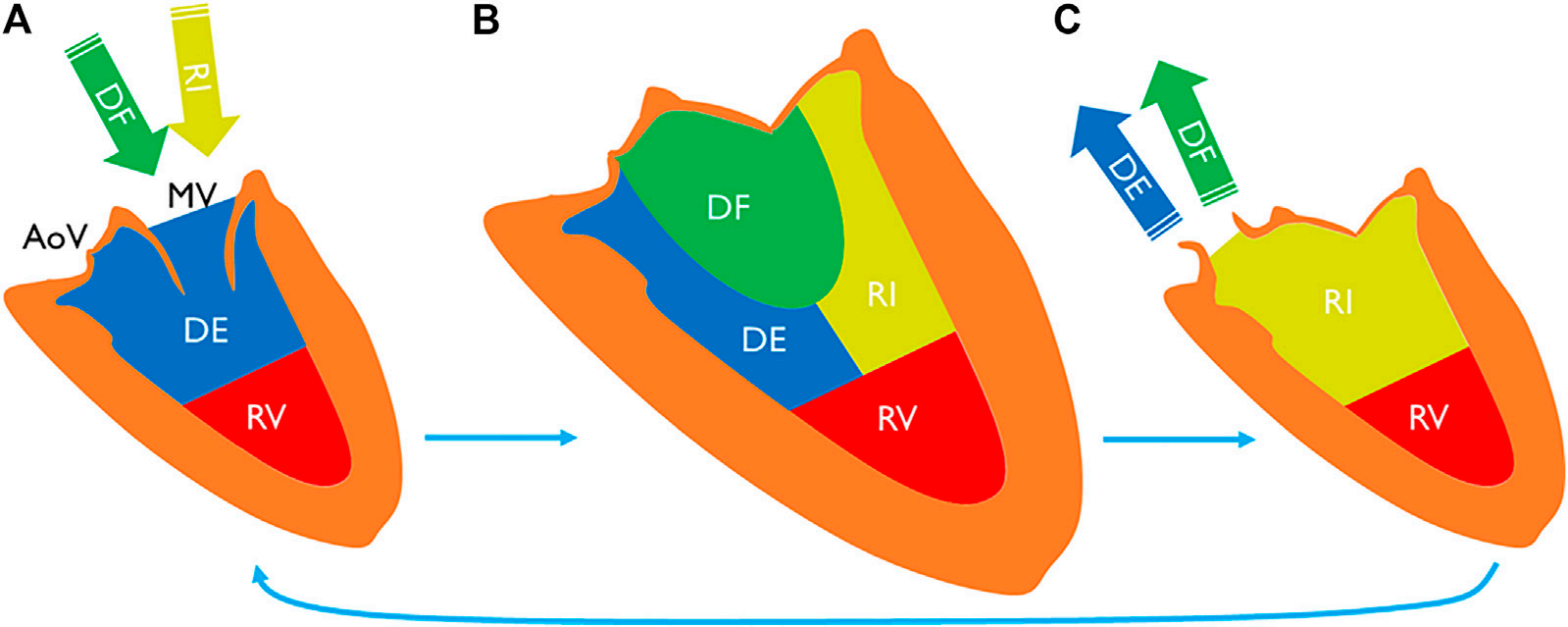
LV blood volume **(A)** at the beginning of diastole, **(B)** at the end-diastole, and **(C)** at the end-systole. End-diastolic volume can be separated into four components. Direct flow is a part of inflow during diastole **(A** to **B)**, exiting the chamber during systole **(B** to **C)**. Retained inflow is another part that remains in the chamber at the end-systole **(C)**. Delayed ejection is a part of LV volume at the beginning of diastole **(A)** that exits during the cycle. Residual volume stays in the chamber for more than one cycle. MV indicates mitral valve; AoV, aortic valve; DF, direct flow; RI, retained inflow; DE, delayed ejection; and RV, residual volume.

To date, LV flow component analysis has been largely used to study hemodynamic efficiencies of healthy subjects and patients with heart failure (Eriksson et al., 2010; Stoll et al., 2019). In this method, blood that flows through the LV during a heart cycle is separated into four components (Figure 1) (Bolger et al., 2007). Direct flow is the blood that enters and leaves the LV during a cycle. Retained inflow is the blood that enters the LV during diastole and stays in at the end of systole. Delayed ejection is that retained blood at the beginning of diastole, which leaves the LV during systole. Residual volume is that blood stays in the LV for more than one cycle. This method has been applied in AF referral populations to study LV function recovery after cardioversion (Karlsson et al., 2019); however, direct comparison of these novel hemodynamic markers has not been studied between AF patients versus healthy subjects. Given the expanding clinical burden of AF patients’ heart failure with preserved ejection fraction (HFpEF) among patients (Savarese and Lund, 2017), a detailed understanding of abnormalities in LV flow distribution within this patient population is strongly justified.

In this study, we aimed to identify the presence of LV hemodynamic inefficiencies in patients with PAF and normal systolic function using flow distribution based on 4-D flow component analysis. We hypothesized that the fraction of direct flow to the total end-diastolic volume is reduced in patients with PAF compared with the healthy controls.

## Methods

### Patient Population

A total of 50 patients with PAF with normal systolic function (age = 56 ± 12, female = 16, scanned between July 2017 and August 2019) and 30 healthy controls (age = 50 ± 8, female = 10, scanned between August 2017 and December 2020) were retrospectively selected from our local database. The PAF initial cohort included 103 patients and healthy control cohort 35 subjects. Patients and controls were selected to match by age given that the prevalence of AF increase in parallel with the aging of the population (Lakshminarayan et al., 2006). All patients were enrolled in the Cardiovascular Imaging Registry of Calgary (CIROC) at the time of clinical referral for cardiac MRI prior to consideration of pulmonary vein ablation for PAF. CHA_2_DS_2_-VASc score of PAF patients was calculated to assess stroke risk level, using patient medical history prior to MRI examination (Pamukcu et al., 2010). A commercial software (cardioDI™, Cohesic Inc., Calgary, Canada) was used to coordinate routine capture of patient informed consent and health questionnaires and for standardized collection of MRI-related variables. Healthy control subjects were older than 18 years of age with no cardiovascular disease and no history of hypertension or diabetes. Patients with significant mitral or aortic valve disease and cases with inappropriate/incomplete image reconstruction were not included. Healthy control screening was performed by a certified research nurse from our institution. The study was approved by the University of Calgary’s Conjoint Health Research Ethics Board, and all subjects provided written informed consent. All research activities were performed in accordance with the Declaration of Helsinki.

### MRI Data Acquisition

Patients and healthy subjects were required to be in sinus rhythm at the time of CMR imaging. All subjects underwent an identical standardized MRI protocol using 3 T MR scanners Skyra/Prisma (Siemens, Erlangen, Germany) inclusive of standard multi-planar steady-state free-precession (SSFP) cine imaging in four-, three-, and two-chamber, short-axis of the LV at end-expiration, and 3-D magnetic resonance angiography (MRA) of the LA using administration of 0.2 mmol/kg gadolinium contrast (Gadovist^®^, Bayer Inc., Mississauga, Ontario, Canada). Approximately 5–10 min following contrast injection, 4-D flow MRI was performed using a prospectively triggered sequence with respiratory navigator-based gating (WIP 785A; n = 68, WIP 1106; n = 12). Whole heart 4-D flow MRI was added at the end of the protocol for the standard cardiac assessment of 3-D intra-cardiac hemodynamics. 4-D flow imaging parameters were as follows: gating = prospective, flip angle = 15°, FOV = 200–420 mm × 248–368 mm, spatial resolution = 2.0–3.5 × 2.0–3.5 × 2.5–3.5 mm; temporal resolution = 25–35 ms, 25–30 phases, and velocity sensitivity = 150–200 cm/s. Total acquisition time varies between 8 and 12 min, depending on heart rate and respiratory navigator efficiency.

### MRI Data Processing and Analysis

Volumetric chamber measurements were performed from standard ECG-gated cine images using commercial software (cvi42 v5.11, Circle Cardiovascular Imaging Inc., Calgary, Canada). Short axis cine images were used to obtain LV end-diastolic volume (EDV) (ml), LV end-systolic volume (ESV) (ml), LV stroke volume (SV) (ml), LV mass (g), LV cardiac output (CO) (L/min), LVEDV indexed to BSA (LVEDV/BSA) (ml/m^2^), LVESV indexed to BSA (LVESV/BSA) (ml/m^2^), LV mass indexed to BSA (LV mass/BSA) (g/m^2^), and LV ejection fraction (LVEF) (%). LA volume (ml) and LA volume indexed to BSA (ml/m^2^) were measured using the two- and four-chamber views using the biplane area-length method.

**FIGURE 2:**
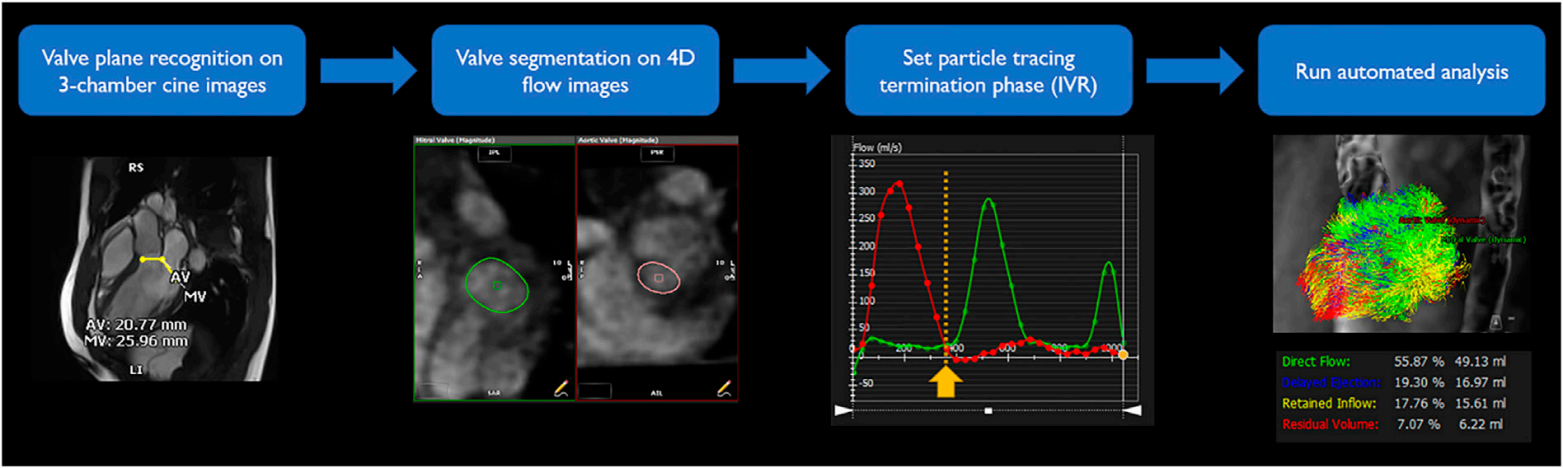
LV flow component analysis workflow. To obtain segmentation of entrance and exit of the LV, valve plane was recognized on three-chamber cine images. Exact shape of the valves was segmented on 4-D flow images for each time frame. Iso-volumetric relaxation phase was identified from time-flow graph, minimizing both flows through the valves. From and to the selected phase, automated simulation traced particles and divided into four components.

4-D flow imaging was analyzed using a dedicated module from cvi42 v5.11. LV flow component analysis is based on a previously presented and validated method (Eriksson et al., 2010; Eriksson et al., 2011). Automated valve tracking was recently incorporated to improve accuracy and reduce variability (Garcia et al., 2021). All data underwent preprocessing to correct for Maxwell terms, eddy current–induced phase offset, and velocity aliasing if necessary. Then, from the long-axis three-chamber cine image, the locations of the aortic and mitral valve planes were automatically recognized on each frame to constrain 4-D flow analysis (Figure 2). The exact contour of the valves was refined manually on 4-D flow images. *Trans*-valvular flow was calculated throughout the cycle, and the isovolumetric relaxation (IVR) phase identified where both flows were minimized after systole. From each voxel in the LV segmentation an automated pathline was emitted. Pathline particles were created backward and forward in time until the identified end-systole within the velocity field Runge–Kutta method (Butcher, 2007). Overall pathlines represent the entire cardiac cycle particle tracking, pathline positions relative at the time of end-systole relative to the cardiac chambers are used to separate them into four flow components (Figure 1) with both the volume and ratio for each component reported. Flow components in the analyzed heartbeat are defined by direct flow (blood that enters the LV during diastole and leaves the LV during systole), retrained inflow (blood that enters the LV during diastole but does not leave during systole), delayed ejection flow (blood that starts and resides inside the LV during diastole and leaves during systole), and residual volume (blood that resides within the LV, not a component of inflow or ejected volume).

### Statistics

Statistics were analyzed using IBM SPSS Statistics for Windows, version 26.0 (IBM Corp., Armonk, N.Y., United States). Continuous variables were tested by the Shapiro–Wilk test to determine if parameters were normally distributed. If parameters were normally distributed, they were presented as mean ± standard deviation, and the independent-samples *t*-test was performed to compare between the PAF and control groups. When the parameters did not satisfy the normality assumption, they were presented as median with (the first quartile - the third quartile), and Mann–Whitney *U*-test was used. A multiple linear regression was performed to assess dependency of flow distribution variables on demographic and conventional LV parameters. The Pearson method was used to assess the correlation and agreement between parameters. Categorical variables are presented by count and frequency and compared using a chi-square test between two groups. For all analyses, two-tailed tests with *p*-value < 0.05 were considered statistically significant. Propensity score matching was performed to assess baseline confounders and their impact on flow distribution variables.

## Results

### Patient Characteristics

The baseline characteristics of patients and controls are given in Table 1. A significant difference was observed in the mean age of the PAF cohort versus controls (56 ± 12 years vs. 50 ± 8 years, *p* = 0.009). Also, weight and BSA tended to be higher in the PAF group (81.5 kg [72.8–100.0] vs. 76.0 kg [61.0–82.2], *p* = 0.011 and 1.99 m^2^ [1.84–2.21] vs. 1.90 m^2^ [1.71–2.02], *p* = 0.016, respectively). Sex ratio, height, heart rate, and systolic/diastolic blood pressure did not show significant differences. CHA_2_DS_2_-VASc risk score was 0 or 1 in 82% of PAF subjects (Table 2) while five subjects had a score of two, and only three subjects having a score of three. These three latter cases were in two females with age >75 and in a female >65 with hypertension. No patients had score of four or above. Overall, our PAF group mostly consisted of low-risk patients. Standard volume and function measurements of the left ventricle and left atrium are summarized in Table 3. LV EDV indexed to BSA was lower in the PAF cohort than in the controls (76.5 [66.4–87.3] vs. 88.8 [77.4–97.0], *p* = 0.006). There were no other significant differences between the PAF and control groups.

**TABLE 1:** Baseline characteristics.

	Controls (n = 30)	PAF patients (n = 50)	*p*-value
	Mean ± SD, median [range] or count (percent)	Mean ± SD, median [range]or count (percent)	
Age (years)	50 ± 8	56 ± 12	0.009
Sex (female)	10 (33%)	16 (32%)	0.902
Height (m)	1.75 [1.67–1.79]	1.77 [1.70–1.86]	0.062
Weight (kg)	76.0 [61.0–82.2]	81.5 [72.8–100.0]	0.011
BSA (m^2^)	1.90 [1.71–2.02]	1.99 [1.84–2.21]	0.016
HR (bpm)	59 [54–66]	63 [55–79]	0.077
Systolic BP (mmHg)	114 ± 12	116 ± 12	0.389
Diastolic BP (mmHg)	70 [55–74]	69 [63–75]	0.806

**TABLE 2:** Risk score and factors in PAF group.

	PAF (n = 44)
CHA_2_DS_2_-VASc	
0	18 (41%)
1	18 (41%)
2	5 (11%)
3	3 (7%)
Risk factors	
CHF/LV dysfunction	2 (6%)
Hypertension	7 (19%)
Aged 75 or over	1 (3%)
Diabetes	2 (6%)
Stroke	0 (0%)
Vascular disease	1 (3%)
Aged 65–74	10 (28%)
Sex category female	13 (36%)

*CHF, congestive heart failure; LV, left ventricle.

**TABLE 3:** Left ventricular and atrial volumes and function.

	Controls (n = 30)	PAF patients (n = 50)	*p*-value
	Mean ± SD, median [range]	Mean ± SD, median [range]	
LV End-Diastolic Volume (ml)	162.6 ± 27.7	159.7 ± 34.4	0.693
LV End-Diastolic Volume indexed to BSA (ml/m^2^)	88.8 [77.4–97.0]	76.5 [66.4–87.3]	0.006
LV End-Systolic Volume (ml)	62.5 [45.9–75.8]	61.0 [51.5–79.5]	0.531
LV End-Systolic Volume indexed to BSA (ml/m^2^)	33 ± 7.3	31.9 ± 7.7	0.510
LV Mass (g)	103.8 ± 26.1	106.6 ± 27.2	0.658
LV Mass indexed to BSA (g/m^2^)	54.6 ± 9.8	52 ± 10.2	0.267
LV Stroke Volume (ml)	101.2 ± 14.7	94.5 ± 23.7	0.121
LV Cardiac Output (L/min)	5.9 ± 0.9	6.0 ± 1.3	0.852
LV Ejection Fraction (%)	61.2 [58.9–68.1]	60.3 [56.9–63.8]	0.061
LA Volume (ml)	69.4 ± 17.5	80.2 ± 26.4	0.054
LA Volume indexed to BSA (ml/m^2^)	36.8 ± 9.2	39.5 ± 12.4	0.317

### Left Ventricular Flow Distribution

**FIGURE 3:**
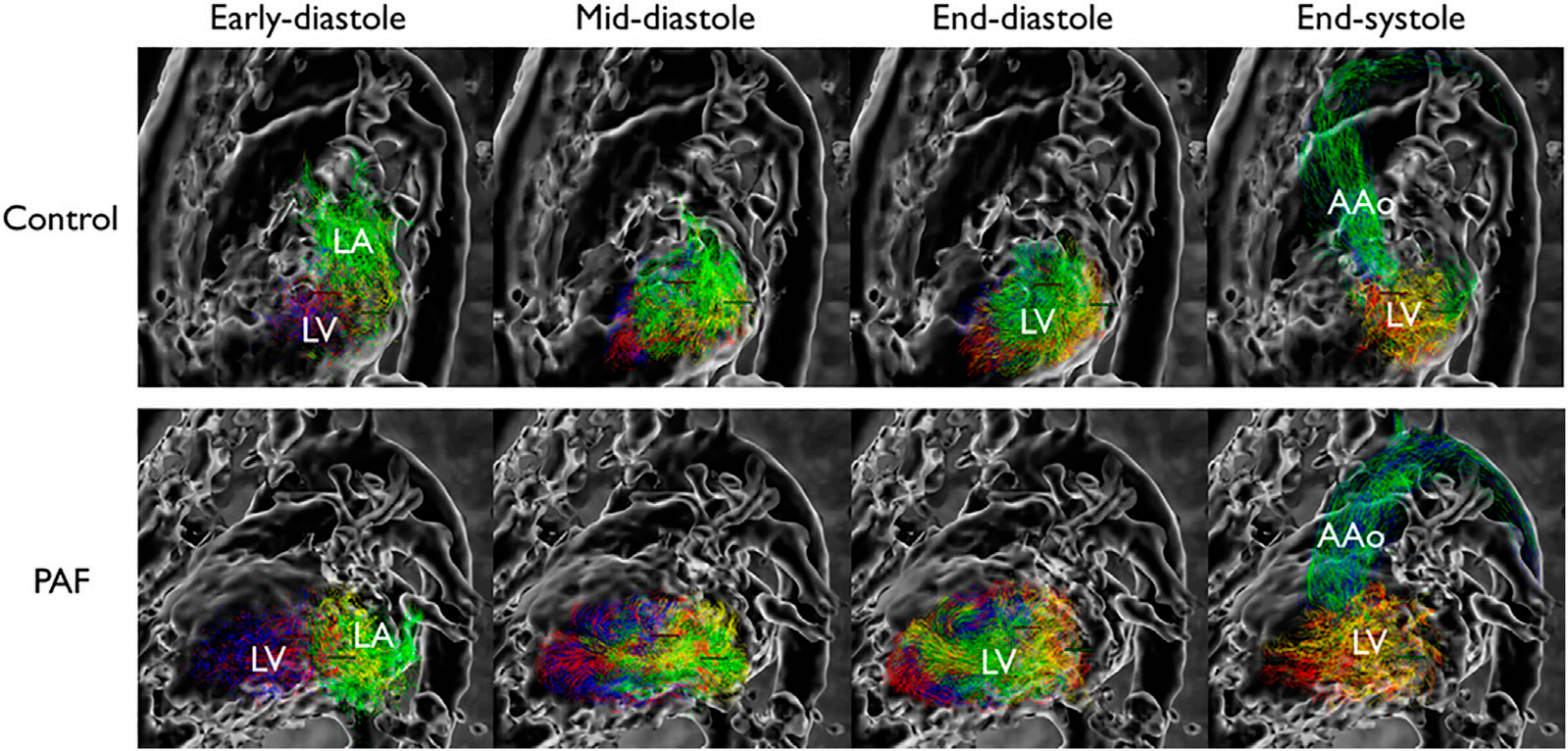
Flow pattern example in left ventricle. Each component is color-coded; green: direct flow, yellow: retained inflow, blue: delayed ejection, red: residual volume. Reduced proportion of direct flow is observed at end-diastolic phase in PAF. LA indicates left atrium; LV, left ventricle; and AAo, ascending aorta.

Examples of LV flow component analysis from controls and PAF patients are shown in Figure 3. Pathlines show direct flow and retained inflow coming into the LV during diastole, followed by outgoing flow consisting of direct flow and delayed ejection during systole. At end-diastole, when all pathlines reside in the LV, each of the different proportions of flow components are uniquely labeled.

**FIGURE 4:**
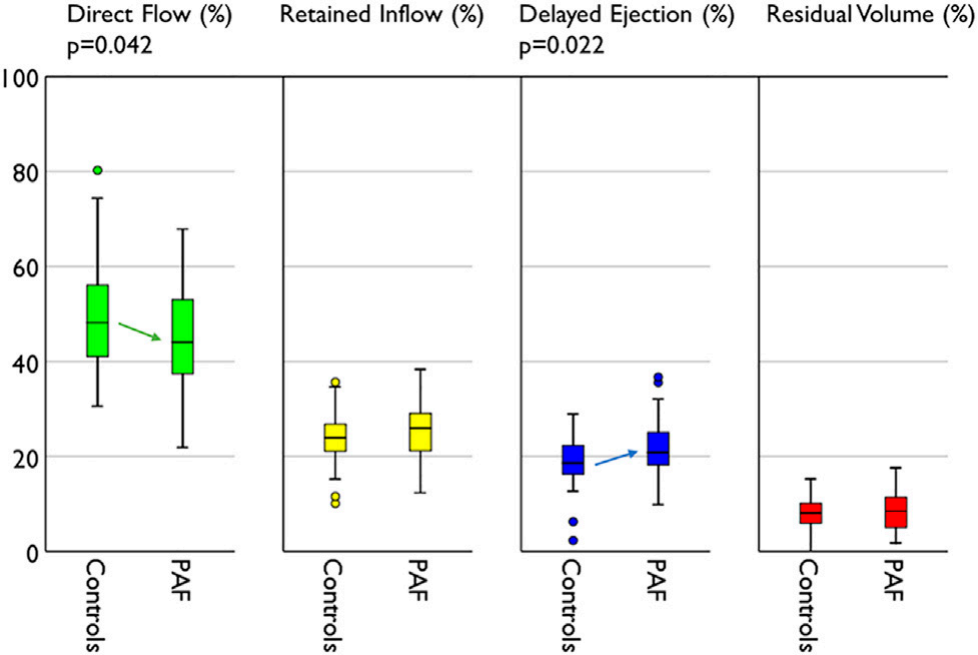
Compare of flow distribution between groups. PAF group represents reduced direct flow and increased delayed ejection compared with controls.

The volume of each flow component and its ratio to the total flow are presented in Table 4. The ratio of direct flow to the total flow was significantly reduced (−11%) in the PAF group compared with controls (44.5 ± 11.2 vs. 50.0 ± 12.2, *p* = 0.042). Delayed ejection was reciprocally increased (+16%) in the PAF group (21.6 ± 5.6 vs. 18.6 ± 5.7, *p* = 0.022) (Figure 4). Observed differences in retained inflow and residual volume were not significant.

**TABLE 4:** Left ventricular flow distribution.

	Controls (n = 30)	PAF patients (n = 50)	*p*-value
Volumes of flow components			
Direct Flow (ml)	37.4 ± 13.4	33.5 ± 13.1	0.204
Retained Inflow (ml)	18.1 ± 7.0	19.8 ± 9.2	0.348
Delayed Ejection (ml)	15.0 ± 6.6	16.5 ± 7.1	0.390
Residual Volume (ml)	6.6 ± 4.1	7.4 ± 5.5	0.517
Ratio of flow components to the total flow			
Direct Flow (%)	50.0 ± 12.2	44.5 ± 11.2	0.042
Retained Inflow (%)	23.5 ± 5.8	25.3 ± 5.5	0.164
Delayed Ejection (%)	18.6 ± 5.7	21.6 ± 5.6	0.022
Residual Volume (%)	8.0 ± 3.8	8.7 ± 4.1	0.450

To evaluate for independent associations of direct flow (reduction) and delayed ejection (elevation) with known AF diagnosis, two separate multivariable models were constructed adjusting for age, sex, BSA, and hypertension. These variables failed to predict direct flow and delayed ejection statistically significantly, F (4, 46) = 0.685, *p* = 0.606, *R* ^2^ = 0.056, and F (4,46) = 1.712, *p* = 0.163, *R* ^2^ = 0.130, respectively.

Correlation with LV function parameters (SV, LVCO, and LVEF) were also evaluated. LVEF was moderately correlated with both direct flow and delayed ejection (r = 0.432, *p* < 0.001 and r = −0.357, *p* = 0.001, respectively).

Propensity score matching considered age, weight, and BSA (Table 1) for further analysis of flow components. After balancing the comparability of the baseline characteristics between groups, only 18 PAF patients were matched to healthy volunteers. There were no statistical differences between propensity score matched cohorts for flow component parameters.

## Discussion

This study compared 4-D flow-derived LV component analysis in patients with PAF and normal LVEF versus healthy controls. Following multivariable adjustment for age, sex, BSA, and hypertension, we identified significant and reciprocal alterations in direct flow (reduced) and delayed ejection (elevated) in patients with AF. This finding provides insights into the unique hemodynamic phenotype of this referral population that may explain their strong predisposition toward HFpEF.

The age difference between study groups is a recognized limitation of this study. Age is one of the most impacting factors on the risk of AF and its complications as well as sex. Nevertheless, correlation analysis between age and flow components demonstrated no significant correlation. This revealed flow component analysis is robust to such an external factor, and therefore, the impact from age difference should be limited.

A certain degree of elevated weight and BSA in the PAF group can be explained by the well-known fact that overweight and obesity are associated with the onset of AF (Frost et al., 2005). LV EDV indexed to BSA was rather low in the PAF group but still within normal range. This can be interpreted as AF burden having not yet impacted LV dilation, so normal LV volume size was normalized by elevated BSA. The similarity observed in other LV and LA volume and function parameters indicate that our enrolled group of PAF subjects were a relatively healthy referral population at an early stage of the disease.

Compared with a prior study evaluating LV component analysis in healthy subjects (Stoll et al., 2018), we observed higher direct flow with reduced residual volumes (50 and 8%, respectively). However, it is important to recognize that the previous study used manual segmentation of the LV volume at only the end-diastolic and end-systolic phases; these are used to constrain flow analysis throughout the cardiac cycle. In our study, both mitral and aortic valve planes were dynamically tracked with iterative, phase-adjusted calculation of blood flow through each valve, which improves flow quantification (Garcia et al., 2021). Although, particle tracing near the LV apex of the heart was reduced in most cases, which may cause underestimation of the residual volume and, in consequence, overestimation of the proportion of direct flow. The latter may be due to the particle tracing algorithm used by the vendor and the low velocities near the apex. A recommended validation is based on comparing inflow vs. outflow for every subject. Further investigation into larger cohorts and the sophisticated algorithm will enable clinical use.

Heart failure (HF) and AF often coexist and have worse prognosis than those with isolated HF or AF (Hindricks et al., 2021). The prevalence of both AF and HF also increases with age. AF can promote HF progression through cardiomyopathy characterized by loss of atrial systole, LA dilation, and rapid irregular ventricular rate affecting LV emptying and filling (Olsson et al., 2006). When EF is preserved, the management is mainly based on symptomatic therapy and treatment for comorbidities. In our study, PAF patients had preserved EF and were in sinus rhythm at the time of the scan. Of interest, DF and DE were significantly different in PAF patients as compared with controls. Both reduction of DF and elevation of DE are markers of altered LV emptying and filling and may be able to early characterize the predisposition to HFpEF.

Propensity score matching assessment considered age, weight, and BSA for further analysis of flow components. A matched cohort was reduced to 18 subjects with no significant differences on flow component parameters. The latter may be due to the matched cohort size and highlights the importance of considering baseline cofounding factors in future studies employing flow component analysis.

## Limitations

Although we demonstrate that demographic factors are not correlated with flow distributions, still the age gap between two groups is a potential cofounding factor. Recruiting enough age-matched controls will resolve the bias in the study groups. Blood pressure measurements were not available in some subjects, especially in the control group. Collecting complete data sets will make sure that this flow distribution has not been affected by external factors. We used commercial software for flow distribution analysis. Although it provided a convenient workflow for quantification of flow components, we could not access the raw simulation results to investigate further. The cause of difference in flow proportion with previous studies was postulated but not confirmed. It is important to remark that flow component analysis is sensitive to degraded preprocessing correction, such as eddy currents, concomitant fields, noise, and aliasing corrections. In addition, quality control of the preprocessed data sets is relevant for the careful identification of aberrant pathlines (Eriksson et al., 2010). Pathline common errors include, but they are not limited to, spatial and temporal resolution, interpolation truncation, and implementation algorithms (Chandler et al., 2015). Implementation of flow analysis by our own will allow access to raw results that reveal whether this method reconstructs LV volume successfully, comparison with manual LV volume segmentation method, and also further study such as energy-based analysis. Propensity score matching findings were limited by the number of matching subjects (n = 18). Based on previous studies, a minimum of 30 subjects is needed to detect differences in flow components between groups assuming a power of 80% and type 1 error of 0.5% (*p* <0 .005) (Eriksson et al., 2010; Eriksson et al., 2011; Kim et al., 2020).

## Conclusion

This study used LV flow component analysis by 4-D flow MRI to demonstrate occult changes in LV flow efficiency in patients with PAF and normal systolic function. Hemodynamic alterations were observed showing reduction in direct flow in the absence of additional markers of adverse LV remodeling, such as LV hypertrophy or chamber dilation. Cofounding baseline factors need to be considered for a more representative assessment of flow components. Expanded study into mechanisms underlying reductions in LV efficiency in this patient population and its relevance to future risk of HFpEF is required.

## Data Availability

The anonymized raw data supporting the conclusion of this article will be made available on request from the corresponding author.
